# The polarity protein Scrib mediates epidermal development and exerts a tumor suppressive function during skin carcinogenesis

**DOI:** 10.1186/s12943-015-0440-z

**Published:** 2015-09-17

**Authors:** Helen B. Pearson, Edwina McGlinn, Toby J. Phesse, Holger Schlüter, Anuratha Srikumar, Nathan J. Gödde, Christina B. Woelwer, Andrew Ryan, Wayne A. Phillips, Matthias Ernst, Pritinder Kaur, Patrick Humbert

**Affiliations:** Peter MacCallum Cancer Centre, St Andrew’s Place, East Melbourne, VIC 3002 Australia; Sir Peter MacCallum Department of Oncology, The University of Melbourne, Parkville, VIC 3010 Australia; EMBL Australia, Australian Regenerative Medicine Institute, Monash University, Clayton, VIC 3800 Australia; Walter and Eliza Hall Institute of Medical Research, Melbourne, VIC 3052 Australia; Present address: Olivia Newton-John Cancer Research Institute and School of Cancer Medicine at La Trobe University, Heidelberg, VIC 3084 Australia; Present address: National Center for Tumor Diseases Heidelberg (NCT), German Cancer Research Centre (DKFZ), 69120 Heidelberg, Germany; TissuPath Laboratories, Mount Waverley, VIC 3149 Australia; Department of Surgery (St. Vincent’s Hospital), The University of Melbourne, Parkville, VIC 3010 Australia; Department of Pathology, The University of Melbourne, Parkville, VIC 3010 Australia; Department of Biochemistry and Molecular Biology, The University of Melbourne, Parkville, VIC 3010 Australia

**Keywords:** Polarity, Scrib, Skin, Carcinogenesis, Permeability barrier

## Abstract

**Background:**

The establishment and maintenance of polarity is vital for embryonic development and loss of polarity is a frequent characteristic of epithelial cancers, however the underlying molecular mechanisms remain unclear. Here, we identify a novel role for the polarity protein Scrib as a mediator of epidermal permeability barrier acquisition, skeletal morphogenesis, and as a potent tumor suppressor in cutaneous carcinogenesis.

**Methods:**

To explore the role of Scrib during epidermal development, we compared the permeability of toluidine blue dye in *wild-type*, *Scrib* heterozygous and *Scrib* KO embryonic epidermis at E16.5, E17.5 and E18.5. Mouse embryos were stained with alcian blue and alizarin red for skeletal analysis. To establish whether Scrib plays a tumor suppressive role during skin tumorigenesis and/or progression, we evaluated an autochthonous mouse model of skin carcinogenesis in the context of Scrib loss. We utilised *Cre-LoxP* technology to conditionally deplete *Scrib* in adult epidermis, since *Scrib KO* embryos are neonatal lethal.

**Results:**

We establish that Scrib perturbs keratinocyte maturation during embryonic development, causing impaired epidermal barrier formation, and that Scrib is required for skeletal morphogenesis in mice. Analysis of conditional transgenic mice deficient for *Scrib* specifically within the epidermis revealed no skin pathologies, indicating that Scrib is dispensable for normal adult epidermal homeostasis. Nevertheless, bi-allelic loss of *Scrib* significantly enhanced tumor multiplicity and progression in an autochthonous model of epidermal carcinogenesis *in vivo*, demonstrating Scrib is an epidermal tumor suppressor. Mechanistically, we show that apoptosis is the critical effector of Scrib tumor suppressor activity during skin carcinogenesis and provide new insight into the function of polarity proteins during DNA damage repair.

**Conclusions:**

For the first time, we provide genetic evidence of a unique link between skin carcinogenesis and loss of the epithelial polarity regulator Scrib, emphasizing that Scrib exerts a wide-spread tumor suppressive function in epithelia.

**Electronic supplementary material:**

The online version of this article (doi:10.1186/s12943-015-0440-z) contains supplementary material, which is available to authorized users.

## Introduction

SCRIB is a large scaffold protein containing 16 leucine-rich repeats (LRRs) and 4 PDZ (PSD95-DLG1-ZO1) protein-interacting domains that interact with Discs large 1–4 (DLG1-4) and Lethal giant larvae (LGL1/2) to form the Scribble complex. The Scribble complex resides along the basolateral membrane of epithelial cells and is concentrated at cell-cell junctions [1–3]. In concert with the polarity program, the Scribble complex engages multiple signal transduction cascades to establish apical-basal and planar cell polarity to regulate key cellular processes including proliferation, apoptosis and differentiation [1–4]. Consequently, loss of epithelial organisation/polarization causes aberrant embryonic morphogenesis [5–11], and is a common feature during epithelial cancer formation and progression [1–4, 7].

*Scrib* is an essential gene in flies and mammals; *scrib* mutant *Drosophila* larvae fail to pupate owing to lethal overgrowth [5] and *Scrib* null mice are neonatal lethal owing to severe neural tube and abdominal wall closure defects [7, 8, 10]. During embryonic development, Scrib is also required for lung morphogenesis [9] and cochlear sterociliary bundle organisation [12], underlining the possibility that Scrib universally mediates epithelial morphogenesis.

Establishment of a functional epidermal permeability barrier (EPB) is essential for post-natal survival of all terrestrial life, preventing dehydration and protecting against physical, chemical and mechanical damage [13–15]. EPB formation occurs during late gestation within the outer layer of the epidermis termed the stratum corneum (SC), which comprises corneocytes (terminally differentiated keratinocytes) surrounded by extracellular lipid lamellae [13–15]. The complex assembly of the EPB involves the provision of lipids and proteins from lamellar granules in the stratum granulosum (SG) to the SC, keratins, cross-linking of envelope proteins (e.g. loricrin and involucrin), and membrane-anchoring proteins (e.g. envoplakin) [13–16]. In addition, tight junctions are also necessary within the SG to mediate paracellular transport and apical-basal polarity for EPB function [17].

Developing a comprehensive molecular understanding of EPB acquisition is paramount for improving our management of common skin disorders associated with an EPB defect, such as psoriasis and dermatitis. Emerging evidence in the literature suggests that aberrant cell polarity may hinder multiple processes required for EPB acquisition. For example, atypical Protein Kinase C (aPKC), which forms the Par3 apical polarity complex with the PAR (partitioning-defective) proteins Par3 and Par6, has been shown to be required for tight junction formation and barrier function *in vitro* [18]. In addition, the planar cell polarity protein Grainyhead-like-3 (Grhl3) and E-cadherin have been previously shown to regulate EPB acquisition by mediating SC formation, cell adhesion and/or extracellular lipid composition in the SG *in vivo* [19–22]. Taken together, these data indicate that an intact polarity network is necessary for EPB assembly, however the true extent of the polarity network’s involvement during EPB acquisition and the molecular mechanisms involved remain elusive.

Mature mammalian skin is comprised of multiple keratinised and stratified layers of polarised squamous epithelial cells and constantly undergoes a high rate of self-renewal. Basal keratinocytes continually proliferate to replace suprabasal keratinocytes that terminally differentiate as they migrate towards the surface, and are finally sloughed off from the dead/flattened cornified layer [23]. Events that cause an imbalance between keratinocyte self-renewal, differentiation and maturation processes result in cutaneous disorders including eczema, psoriasis [24] and skin cancer [20, 25]. Basal cell carcinoma (BCC) and squamous cell carcinoma (SCC) comprise the majority of non-melanoma skin malignancies, and the latter is a major cause of death worldwide owing to insufficient treatment of metastatic disease [26]. Accordingly, there is an urgent need to improve our molecular understanding of cutaneous skin cancers to identify new routes for therapeutic intervention and novel biomarkers.

In keeping with the concept that oncogenesis involves abnormal signalling through pathways that regulate embryonic development, Scrib has been shown to play a tumor suppressive function in multiple epithelial tissues [7, 27–30] and is frequently deregulated and mislocalised in human epithelial cancers [7, 27, 31, 32]. Furthermore, shRNA-mediated *SCRIB* knockdown in a non-tumorigenic human keratinocyte cell line (HaCaT) is reported to be sufficient to increase cell growth, reduce cell-cell contacts and increase invasion *in vitro* [33].

Collectively, this evidence suggests a potential role for Scrib during embryonic development and cancer formation and progression in skin. To directly test this hypothesis *in vivo*, we have characterised the epidermal phenotype of *Scrib*-deficient embryos and adult mice using a conditional transgenic approach. We show that bi-allelic loss of *Scrib* causes a transient delay in the acquisition of the skin permeability barrier at E17.5, which was associated with impaired keratinocyte maturation. Analysis of adult epidermis identified that Scrib is not required for normal murine epidermal homeostasis, but can facilitate tumor initiation and progression in the two-step 7,12-Dimethylbenz(a)anthracene/12-*O*-Tetradecanoylphorbol-13-acetate (DMBA/TPA) skin carcinogenesis model by reducing apoptosis. Concurrently, these data are the first to establish Scrib as a mediator of EPB formation and a potent tumor suppressor during epidermal carcinogenesis and progression.

## Results

### Scribble loss impairs embryonic skin permeability barrier function

**Fig. 1:**
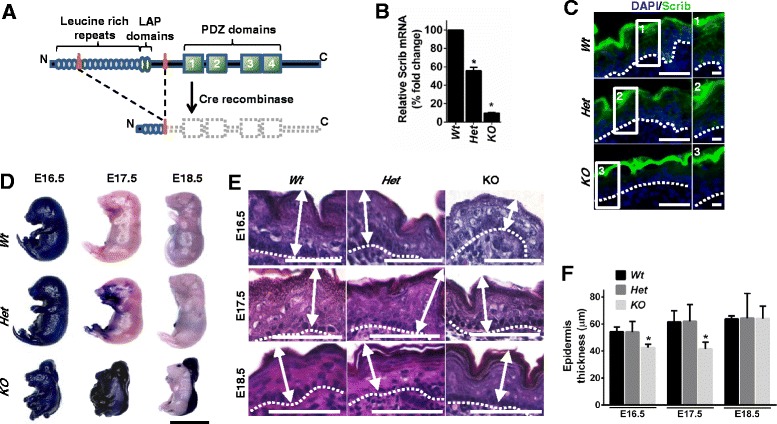
*Scrib KO* embryos display a transient delay in epidermal permeability barrier acquisition. **a** Schematic representation of floxed targeting construct and mutated *Scrib* alleles. LoxP sites are represented by orange triangles. *Cre*-mediated recombination results in excision of exons 4–13, which introduces a frame-shift mutation that truncates the protein. **b** qRT-PCR to detect *Scrib* mRNA in embryonic skin at E16.5 (*n* = 3). Error bars: SD, *p < 0.0001 (unpaired *t*-test). **c** IF to detect Scrib (*green*) and DAPI (*blue*) in embryonic flank epidermis at E18.5 (*n* = 3 per genotype, scale bar = 50 μm. Inserts 1–3 scale bar = 10 μm). Scrib staining was detected along the membrane cortex of epidermal epithelial cells in Wt and Het embryonic epidermis and was absent in *Scrib* KO embryos. All genotypes displayed non-specific epidermal surface staining. **d** Representative images of toluidine blue skin permeability barrier assay (scale bar: 1 cm, *n* = 4–10 per genotype/time point). **e** H&E images of *Wt*, *Het* and *KO* epidermis at E16.5, E17.5 and E18.5, dashed line represents basement membrane, arrows indicate width of epidermis measured (*n* = 8–29 per genotype/time point, scale bar: 50 μm). **f** Quantitation of embryonic epidermal thickness. Error bars: SD, *p ≤ 0.0227 (2-way ANOVA with Tukey correction), *n* = 3 per genotype at each time point

To determine the role of Scrib during epidermal embryonic development, we initially examined the flank epidermis from viable wild-type (*Wt*) and *Scrib*^*+/−*^ (*Het*) embryos compared to neonatal lethal *Scrib*^*-/-*^ knockout (*KO*) embryos at E16.5, E17.5 and E18.5 (*n* = 8–29 per genotype and time point). Germline Cre-deleter driven recombination results in the systemic excision of a LoxP site flanking exons 4–13 of the *Scrib* gene, resulting in a frame-shift mutation and premature truncation of the protein (Fig. 1a). The remaining N-terminal product (approximately 100aa) has no known function. Quantitative real-time PCR (qRT-PCR) analysis confirmed a significant reduction in *Scrib* mRNA transcript expression levels in *Het* and *KO* embryonic flank skin compared to *Wt* littermates at E16.5 (Fig. 1b). Immunofluorescence (IF) to detect Scrib protein revealed uniform membranous staining in *Wt* and *Het* embryonic epidermal cells that is absent in *KO* littermates at E18.5 (Fig. 1c).

To establish whether Scrib is required for EPB acquisition, we assessed the penetration of toluidine blue dye in *Scrib*-deficient embryonic epidermis at several stages of development (Fig. 1d, *n* = 4–10 per genotype/time point). At E16.5, *Wt* and *Scrib*-deficient embryos displayed global dye penetration, consistent with previous work demonstrating that permeability barrier formation begins at this time point, but is not yet fully established [34]. By E17.5, *Wt* and *Het* embryos are resistant to toluidine blue permeation, reflecting SC formation and a functional EPB. In contrast, *Scrib KO* embryos displayed intense blue staining at E17.5, suggesting impaired barrier formation. Nevertheless, by E18.5 *Scrib KO* embryos did not allow dye penetration (except in exposed neural tube and eye) (Fig. 1d). Taken together, our findings indicate that *Scrib* deletion causes a transient delay in epidermal barrier acquisition at E17.5. Consistent with this, histological analysis revealed that Scrib *KO* flank skin was markedly thinner (Fig. 1e), and quantitation revealed a significant reduction in epidermal thickness at E16.5 and E17.5 compared to *Wt* and *Het* embryos that was recovered at E18.5 (Fig. 1f).

**Fig. 2:**
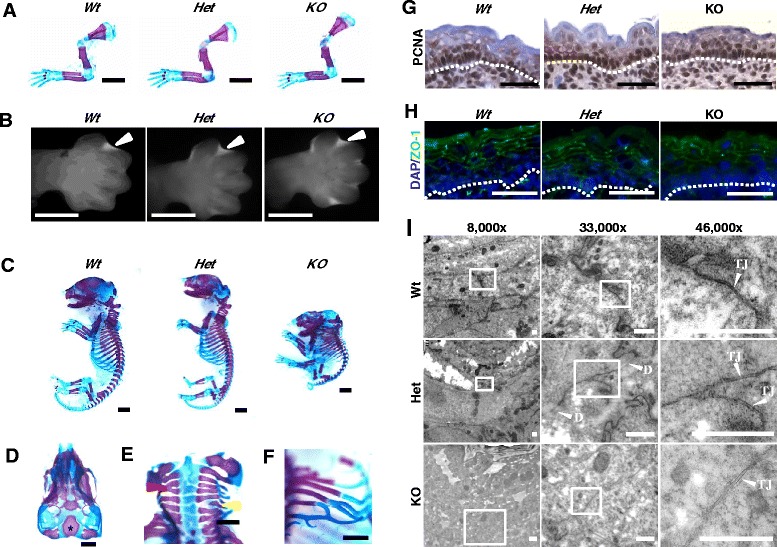
*Scrib KO* embryos display normal developmental timing, proliferation and tight junction distribution. **a** Analysis of forelimb skeletal morphology following alcian blue and alizarin red staining at E17.5. No obvious developmental delay was observed in *Scrib KO* forelimbs when compared with *Wt* or *Het* littermates (scale bar = 2 mm, *n* = 3–6). **b** At E13.5, the characteristic pattern of interdigital apoptosis as revealed by Lysotracker incorporation (*white arrowheads*) indicates no obvious developmental delay in *Scrib KO* embryos when compared with *Wt* or *Het* littermates (scale bar = 1 mm, *n* = 3). **c** Whole E17.5 embryo skeletal analysis (scale bar: 5 mm, *n* = 3–6) reveals extensive dysmorphology in *Scrib KO* embryos, including (**d**) loss of dorsal cranial bones such that the basi-occipital bone (asterix) visible in dorsal view, (**e**) vertebral fusion (**e**, *red arrowhead*), hemivertebra (**e**, *yellow arrowhead*) and (**f**) distal rib fusion . Scale bar in d-f = 1 mm, *n* = 3. **g** IHC to detect PCNA in *Scrib Wt*, *Het* and *KO* embryonic epidermis at E16.5 (scale bar: 50 μm, *n* = 3, dashed line represents basement membrane). **h** IF images to detect ZO-1 (*green*) in *Scrib Wt*, *Het* and *KO* embryonic epidermis at E17.5 (scale bar = 50 μm, DAPI = blue, *n* = 3, dashed line represents basement membrane). **i** Tight junction ultrastructure analysis within the granular layer of *Scrib Wt*, *Het* and *KO* embryonic epidermis at E17.5 at 8,000x, 33,000x and 46,000x magnification (scale bar = 0.5 μm, TJ = tight junction and D = desmosome, *n* = 3–6). Scrib *Wt*, *Het* and *KO* embryos displayed a similar frequency of TJs with normal morphology within the SG

To investigate whether the delay in skin barrier formation we observe in *Scrib KO* embryos was simply due to a generalised delay in developmental timing, we analysed formation of the forelimb. Limb morphogenesis is highly stereotyped and as such, is commonly used as an indicator of developmental stage. Staining of E17.5 forelimbs with alcian blue and alizarin red, to detect cartilage and bone respectively, revealed no obvious difference in bone size, morphology or pattern of ossification in *KO* forelimbs compared to *Wt* or *Het* littermates (Fig. 2a; *n* = 3–6). At early stages of forelimb development, characteristic digit morphology and the dynamic pattern of interdigital apoptosis were unchanged in E13.5 *KO* embryos when compared to *Wt* or *Het* littermate embryos (Fig. 2b; *n* = 3). Together, these data indicate that a global delay in developmental timing is not responsible for the observed defects we see in skin barrier formation, and suggest that Scrib plays a specific role in regulating epidermal development. Nevertheless, although these data strongly suggest that the transient delay in EPB formation observed in *Scrib* KO embryos is most likely not a consequence of systemic abnormal development, we cannot completely exclude this possibility.

Interestingly, staining for bone and cartilage in *Scrib KO* embryos at E17.5 revealed widespread dysregulation of skeletal morphogenesis outside of the limb that to our knowledge has not been reported previously (Fig. 2c; *n* = 3–6). *Scrib KO* embryos were not only smaller, but displayed loss of dorsal cranial bones, hemivertebra and widespread proximal and distal rib fusions (Fig. 2d-f) resembling Chuzhoi mutant mouse embryos that harbour a mutation in the polarity gene *Ptk7* [35] and Dvl2^−/−^ mouse embryos [36], further illustrating the importance of this network during development.

Although we observe multiple developmental and skeletal abnormalities in *Scrib KO* embryos, our analysis of interdigital apoptosis and limb morphogenesis strongly indicates that developmental timing is not broadly disrupted. Thus, we reasoned that the transient delay in epidermal barrier acquisition might reflect impaired proliferation, Tight Junction (TJ) formation and/or keratinocyte maturation. To test this, we first analysed proliferation by performing immunohistochemistry (IHC) to detect the proliferation marker PCNA at E16.5, E17.5 and E18.5 (Fig. 2 g and data not shown, *n* = 3 per genotype/time-point). Remarkably, *Wt* and *Scrib-*deficient embryonic epidermis displayed a similar number of PCNA-positive cells, indicating that Scrib loss does not cause a transient delay in EPB acquisition by reducing proliferation.

IF to detect the TJ protein ZO-1 revealed that *Scrib* Wt, Het and KO embryonic epidermis display comparable, uniform membranous ZO-1 staining (Fig. 2 h, *n* = 3), indicating that impaired TJ formation is not responsible for the observed delay in EPB formation in *Scrib KO* embryos. In support, ultrastructure analysis of *Scrib KO* embryonic flank epidermis revealed normal TJ morphology (e.g. kissing points) that were comparable to *Wt* and *Het* littermates at E17.5 (Fig. 2i, *n* = 3–6). Furthermore, we did not observe any changes in the distribution of the apical-basal polarity protein Dlg or the Adheren’s Junction (AJ) component and polarity protein E-cadherin (Additional file 1: Figure S1, *n* = 3). Collectively, these data suggest that delayed EPB formation in *Scrib* KO embryos is not the result of aberrant polarity or cell-cell adhesions.

**Fig. 3:**
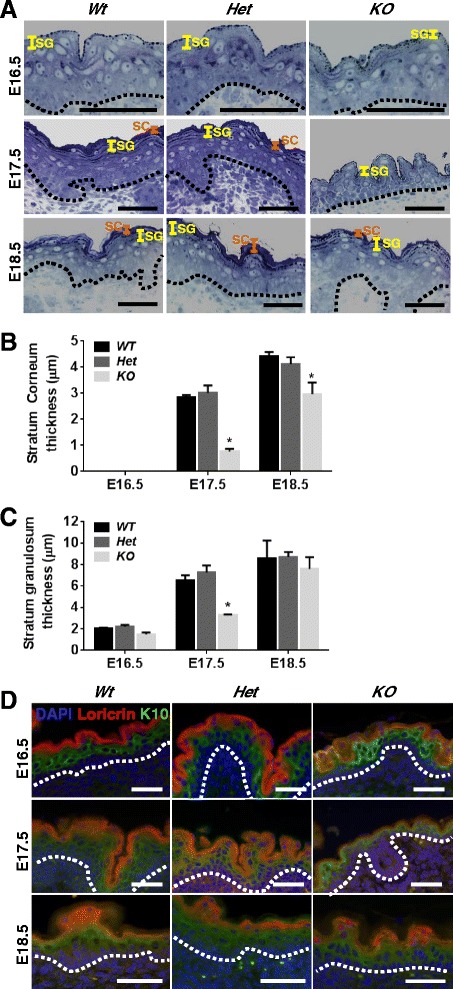
*Scrib KO* embryos display a transient delay in keratinocyte maturation. **a** Toluidine blue stained ultramicrotome sections of *Scrib* Wt, Het and KO embryonic flank epidermis at E16.5, E17.5 and E18.5 indicating stratum corneum (SC, *orange*) and stratum granulosum (SG, *yellow*) thickness (scale bar = 50 μm, dashed line represents basement membrane, *n* = 3). Quantitation of stratum corneum (**b**) and stratum granulosum (**c**) thickness in *Scrib* Wt, Het and KO embryonic flank epidermis at E16.5, E17.5 and E18.5 (*P ≤ 0.0066, two-way ANOVA with Tukey correction, error bars = SD, *n* = 3). **d** IF to detect K10 (*green*), loricrin (*red*) and DAPI (*blue*) in *Scrib* Wt, Het and KO embryonic flank epidermis at E16.5, E17.5 and E18.5 (scale bar = 100 μm, dashed line represents basement membrane, *n* = 3)

To assess whether Scrib loss disrupts keratinocyte maturation, we measured the thickness of both the SC and the SG in *Scrib* Wt, Het and KO epidermis at E16.5, E17.5 and E18.5. We show that *Scrib* KO embryos display a significant reduction in SC and SG thickness at E17.5 compared to Wt and Het littermates (Fig. 3a-c, *n* = 3). Since SG and SC thickness is restored and partially recovered respectively in *Scrib KO* epidermis at E18.5, these data suggest that Scrib loss perturbs keratinocyte maturation to delay EPB formation. In support, co-staining for the spinous layer cytoskeletal protein cytokeratin 10 (K10) and the SG component loricrin, revealed that at E16.5 and E17.5, *Scrib KO* epidermis displays a diminished SC and limited SG formation compared to *Wt* and *Het* littermates, which recovered at E18.5 (Fig. 3d, *n* = 3).

### Scrib is not essential for the maintenance of epidermal tissue homeostasis

**Fig. 4:**
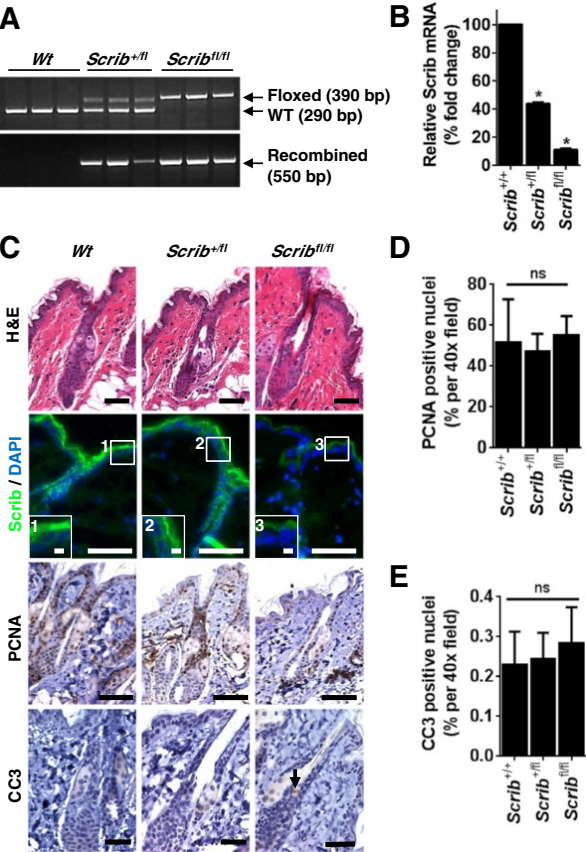
Scrib is dispensable for the maintenance of normal adult epidermal homeostasis. **a** PCR analysis of genomic DNA to detect Wt (290 bp), floxed (390 bp) and recombined (550 bp) *Scrib* alleles in *Scrib* ^*+/+*^, *Scrib* ^*+/fl*^ and *Scrib* ^*fl/fl*^ adult dorsal epidermis (*n* = 3). **b** qRT-PCR for *Scrib* mRNA confirmed a significant reduction in *Scrib* ^*+/fl*^ and *Scrib* ^*fl/fl*^ adult dorsal epidermis compared to *Scrib* ^*+/+*^ controls (*p ≤ 0.0001, unpaired *t*-test, *n* = 3). **c** Representative H&E (*n* = 10), Scrib IF (Scrib = green, DAPI = blue), PCNA IHC and CC3 IHC images of *Scrib* ^*+/+*^, *Scrib* ^*+/fl*^ and *Scrib* ^*fl/fl*^ adult dorsal epidermis (*n* = 3). Scale bar = 50 μm (insert 1–3: scale bar = 10 μm). Arrow represents positive CC3 staining. Quantitation of nuclear PCNA (**d**) and CC3 (**e**) positive cells in adult dorsal epidermis shows no significant difference in *Scrib-*deficient epidermis compared to *Scrib* ^*+/+*^ controls (*p* ≥ 0.6188, unpaired *t*-test, *n* = 3). Adult mice were 100 day old. Error bars = SD

Given that Scrib facilitates EPB acquisition during development, we next analysed whether Scrib functions to maintain epidermal homeostasis in adult mice. As *Scrib KO* mice are neonatal lethal, we crossed *K14Cre(*Δ*neo)* mice [37] with *Scrib* floxed mice [7] to specifically delete *Scrib* in the entire epidermis after birth. Cohorts of *K14Cre*^*+/−*^*;Scrib*^*+/+*^, *K14Cre*^*+/−*^*;Scrib*^*+/fl*^ or *K14Cre*^*+/−*^*;Scrib*^*fl/fl*^ mice (hereafter denoted *Scrib*^*+/+*^, *Scrib*^*+/fl*^, *Scrib*^*fl/fl*^ respectively) were generated and aged to 100 days (*n* = 10). PCR analysis of genomic DNA isolated from dorsal skin confirmed recombination of the *Scrib*^*+/fl*^ and *Scrib*^*fl/fl*^ alleles in the epidermis, while only the *Wt* allele was present in control animals (Fig. 4a). QRT-PCR to detect *Scrib* confirmed that mRNA transcript levels were significantly decreased by 56.3 % (±1.92 SD) and 89.0 % (±1.84 SD) in *Scrib*^*+/fl*^ and *Scrib*^*fl/fl*^ mice respectively (Fig. 4b). Histological analysis of adult epidermis at 100 days revealed no obvious phenotypic difference in *Scrib*^*+/+*^, *Scrib*^*+/fl*^, *Scrib*^*fl/fl*^ epidermis, despite the absence of Scrib protein in *Scrib*^*fl/fl*^ epidermal cells, determined by IF staining (Fig. 4c). *Scrib*^+/+^ and *Scrib*^*+/fl*^ mice showed strong, continuous Scrib staining within the membrane cortex of epidermal cells. Together, these findings indicate that Scrib is dispensable for the maintenance of adult epidermal tissue homeostasis.

To confirm Scrib is not required for normal adult skin homeostasis, we first performed IHC for PCNA, to visualise proliferating cells, and Cleaved Caspase-3 (CC3) to visualise apoptotic cells. Enumeration of these IHC identified that there was no significant difference in the number of proliferating or apoptotic cells in the epidermis of *Scrib*^+/+^, *Scrib*^*+/f*^^l^ and *Scrib*^*fl/fl*^ mice (Fig. 4c-e) at 100 days (*n* = 3). In support, qRT-PCR to detect mRNA expression of the stem cell gene *Lgr5* revealed no significant difference in transcript levels in *Scrib*^+/+^, *Scrib*^*+/f*^^*l*^ and *Scrib*^*fl/fl*^ epidermis (Additional file 2: Figure S2A, *n* = 3). Cytokeratin 6 (K6) is detected in the interfollicular epidermis only during hyperproliferative conditions, including psoriasis [38] and wound healing [39], and thus can be used as a surrogate marker of aberrant homeostasis in the epidermis. IHC to detect cytokeratin 6 (K6) confirmed that K6 was restricted to the hair follicles in *Scrib*^*+/+*^ and *Scrib*-deficient epidermis (Additional file 2: Figure S2B, *n* = 3), indicative of normal homeostatic conditions [38]. Finally, we investigated differentiation in the epidermis by performing co-IF staining for basal (K5) and suprabasal (K10) differentiation markers, which revealed no difference between *Scrib*^+/+^, *Scrib*^*+/f*^^*l*^ and *Scrib*^*fl/fl*^ adult epidermis (Additional file 2: Figure S2C, *n* = 3). Importantly, skin pathologies were not observed in aged *Scrib*^+/+^, *Scrib*^*+/f*^^*l*^ and *Scrib*^*fl/fl*^ mice (400 d, *n* = 10, data not shown). Collectively, these data indicate that Scrib is not essential for murine adult epidermal homeostasis.

### Scrib suppresses tumor formation during DMBA/TPA-induced skin carcinogenesis

We have previously shown that conditional homozygous deletion of *Scrib* mediated by *Probasin-* and *MMTV-*driven *Cre*-recombinase expression causes low-grade prostate intraepithelial neoplasia and mammary gland dysplasia respectively in mice, establishing that *Scrib* is a mammalian tumor suppressor gene [7, 30]. To examine the role of Scrib during epidermal tumor initiation/progression, we used the well-characterised DMBA/TPA two-step chemical skin carcinogenesis model [40, 41]. DMBA invariably induces *HRas* activating mutations, while TPA promotes tumor growth by deregulating several signaling networks, including the PKC-Ras/MAPK cascade that mediates proliferation, differentiation and inflammation [42–44].

**Fig. 5:**
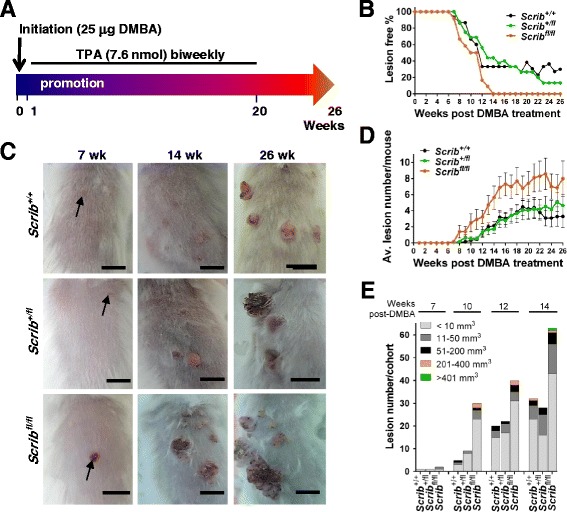
Scrib deficiency facilitates DMBA/TPA-induced epidermal lesion growth. **a** Diagram illustrating long-term two-step DMBA/TPA carcinogenesis approach initiated at 8 weeks of age. **b** Lesion-free percentage, (**c**) representative photographs of dorsal skin at 7, 14 and 26 weeks post-DMBA (scale bar: 1 cm, arrows indicate lesion onset), (**d**) lesion multiplicity plot (error bars = SEM) and (**e**) histogram to illustrate volume distribution of DMBA/TPA-induced epidermal lesions in *Scrib* ^*+/+*^, *Scrib* ^*+/fl*^ and *Scrib* ^*fl/fl*^ mice (*n* = 12–16)

Cohorts of *Scrib*^*+/+*^, *Scrib*^*+/fl*^ and *Scrib*^*fl/fl*^ mice were generated (*n* = 12–16; mixed gender) and underwent long-term DMBA/TPA treatment at 8 weeks of age (Fig. 5a). Weekly examination of dorsal epidermis revealed that papillomagenesis initiated 7 weeks post-DMBA in *Scrib*^*+/+*^, *Scrib*^*+/fl*^ and *Scrib*^*fl/fl*^ mice (Fig. 5b and c), indicating that Scrib loss does not disturb the latency of DMBA/TPA-induced epidermal lesions. Notably, the entire *Scrib*^*fl/fl*^ cohort had developed epidermal lesions at 14 weeks post-DMBA, while 43.7 % and 33.3 % of *Scrib*^*+/+*^ and *Scrib*^*+/fl*^ cohorts respectively were tumor free (Fig. 5b). Indeed, 30.0 % of *Scrib*^*+/+*^ and 13.3 % of *Scrib*^*+/fl*^ mice remained tumor-free during the course of the experiment (Fig. 5b), however overall survival was not significantly different between genotypes (Additional file 3: Figure S3A). qRT-PCR of RNA isolated from epidermal lesions and IF to detect Scrib confirmed *Scrib* mRNA is significantly reduced in *Scrib*^*+/fl*^ and *Scrib*^*fl/fl*^ lesions compared to *Scrib*^*+/+*^ controls, and that Scrib protein is uniformly distributed at the membrane cortex in *Scrib*^*+/+*^ and *Scrib*^*+/fl*^ DMBA/TPA lesions, but negligible in *Scrib*^*fl/fl*^ mice (Additional file 3: Figure S3B and C; *n* = 3). These data demonstrate that Scrib confers a tumor suppressive role during DMBA/TPA-induced skin carcinogenesis.

Quantitation of lesion multiplicity revealed that the incidence of DMBA/TPA-induced lesions is increased in *Scrib*^*fl/fl*^ mice compared to *Scrib*^*+/+*^ and *Scrib*^*+/fl*^ mice (Fig. 5d). In addition, half of the *Scrib*^*fl/fl*^ cohort (6/12) developed lesions that reached our ethical size limit and were sacrificed prior to 26 weeks post-DMBA, compared to only 20 % (3/15) and 25 % (4/16) of the *Scrib*^*+/+*^ and *Scrib*^*+/fl*^ cohorts respectively (Additional file 3: Figure S3A). *Scrib*^*fl/fl*^ mice also developed larger DMBA/TPA-induced lesions compared to *Scrib*^*+/+*^ and *Scrib*^*+/fl*^ mice at comparable time-points (Fig. 5e; 7, 10, 12 and 14-weeks post DMBA treatment shown). Together, these data indicate that bi-allelic loss of *Scrib* sensitises the epidermis to papillomagenesis and facilitates their growth.

### Scrib loss facilitates invasive SCC progression in DMBA/TPA-induced skin carcinogenesis

**Fig. 6:**
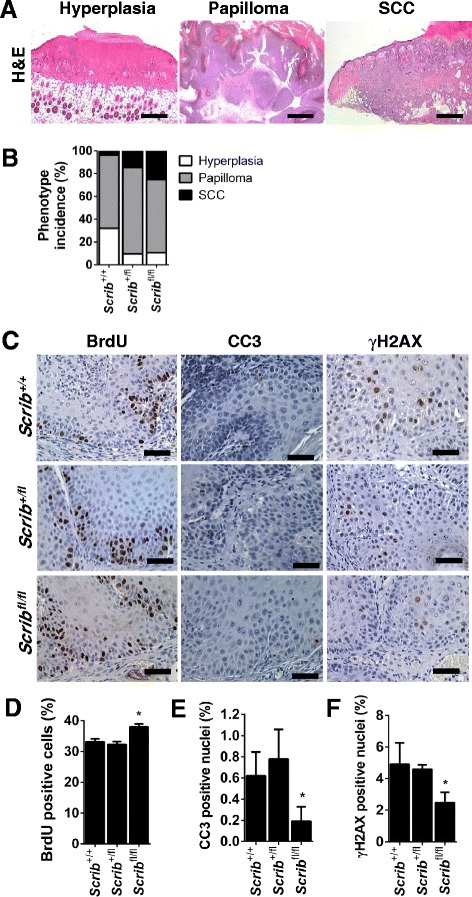
Scrib depletion promotes DMBA/TPA-induced epidermal tumor progression. **a** Representative H&E images of mouse epidermis displaying cutaneous hyperplasia, benign papilloma formation and invasive SCC following long-term DMBA/TPA treatment (scale bar = 1 mm). **b** Percentage phenotype incidence at end-point (*P* = 0.0117, two-way ANOVA, *n* = 12–16) in *Scrib* ^*+/+*^ *, Scrib* ^*+/fl*^ and *Scrib* ^*fl/fl*^ mice that have undergone long-term DMBA/TPA treatment. *Scrib* ^*+/+*^ *Scrib* ^*+/fl*^ and *Scrib* ^*fl/fl*^ respective phenotype incidence; hyperplasia (32.1 %, 9.7 % and 10.7 %), benign papillomas (64.2 %, 75.8 %, 64.3 %) and invasive SCC (3.8 %, 14.4 % and 25 %) . **c** IHC images for BrdU, CC3 and γH2AX (scale bar = 50 μm, *n* = 3) and quantitation of the number of (**d**) BrdU, (**e**) CC3 and (**f**) γH2AX positive cells in *Scrib* ^*+/+*^, *Scrib* ^*+/fl*^ and *Scrib* ^*fl/fl*^ mice that have undergone long-term DMBA/TPA treatment (% per 40x magnification field, *p ≤ 0.0486, unpaired *t*-test, error bars = SD, *n* = 3)

Histological analysis of *Scrib*^*+/+*^, *Scrib*^*+/fl*^ and *Scrib*^*fl/fl*^ mice at sacrifice determined that long-term DMBA/TPA treatment causes epidermal hyperplasia, benign papillomas and invasive SCC in *Scrib*^*+/+*^, *Scrib*^*+/fl*^ and *Scrib*^*fl/fl*^ mice (Fig. 6a). While only a single SCC was observed in the *Scrib*^*+/+*^ cohort (3.8 % incidence), *Scrib*^*+/fl*^ and *Scrib*^*fl/fl*^ mice displayed an increased frequency of benign papilloma progression to invasive SCC (14 % and 25 % incidence respectively) (Fig. 6b). Thus, epidermal tumor progression correlates directly with reduced *Scrib* expression.

Next, we assessed the proliferation status of volume-matched (40 – 80 mm^3^) early benign papillomas from all 3 genotypes, to ascertain whether Scrib loss can accelerate DMBA/TPA-induced papilloma growth. IHC to detect BrdU incorporation revealed that bi-allelic *Scrib* loss causes a small, yet significant increase in the number of mitotic cells (37.3 % ± 1.754 SD) compared to *Scrib*^*+/fl*^ (32.3 % ± 1.742 SD) or *Scrib*^*+/+*^ (33.1 % ± 0.991 SD) papillomas (Fig. 6c and d). Thus, elevated proliferation contributes to the observed increased in DMBA/TPA epidermal tumor volume in *Scrib*^*fl/fl*^ mice.

Given that DMBA initiation invariably causes activating *Ras* mutations in murine epidermis [45], these findings support previous work demonstrating that Scrib loss cooperates with *Ras* oncogenic activation to promote progression and/or invasion [7, 29, 30, 46]. However, we did not observe any significant changes in the number of p-ERK positive nuclei in DMBA/TPA-induced size-matched benign papillomas from *Scrib*^*+/+*^, *Scrib*^*+/fl*^ and *Scrib*^*fl/fl*^ (Additional file 3: Figure S3D and E), suggesting that the increased tumour phenotype observed upon deletion of *Scrib* is unlikely to reflect changes in Ras/MAPK signalling.

Of note, we did not detect any alteration in the distribution of the tight-junction protein ZO-1, or the AJ component E-cadherin (Additional file 3: Figure S3F, *n* = 3), that has been previously shown to co-localise with Scrib to regulate E-cadherin-mediated cell-cell adhesion [47]. These data suggest that Scrib does not alter cell-cell adhesion to facilitate DMBA/TPA progression.

Papillomas developed significantly faster in *Scrib*^*fl/fl*^ mice suggesting that loss of Scrib may provide a tumor promoting environment in the skin. To investigate if Scrib loss promotes the survival of transformed cells in the DMBA/TPA model, we performed IHC to detect CC3 in volume-matched early benign papillomas expressing either *Scrib*^*+/+*^, *Scrib*^*+/fl*^ or *Scrib*^*fl/fl*^ alleles (Fig. 6c). Immunostaining quantitation revealed that the number of CC3 positive cells in *Scrib*^*fl/fl*^ papillomas was significantly reduced compared to *Scrib*^*+/+*^ or *Scrib*^*+/fl*^ papillomas (Fig. 6e). In support, we show that the number of cells positive for the histone variant H2AX phosphorylated at Ser139 (termed γH2AX), previously shown to be associated with ATM- and JNK-dependent apoptosis [48, 49], directly correlates with the number of CC3-positive apoptotic cells (Fig. 6c and f). Taken together, our findings indicate that Scrib deletion promotes DMBA/TPA-induced epidermal lesion growth by enhancing proliferation and survival of transformed cells.

**Fig. 7:**
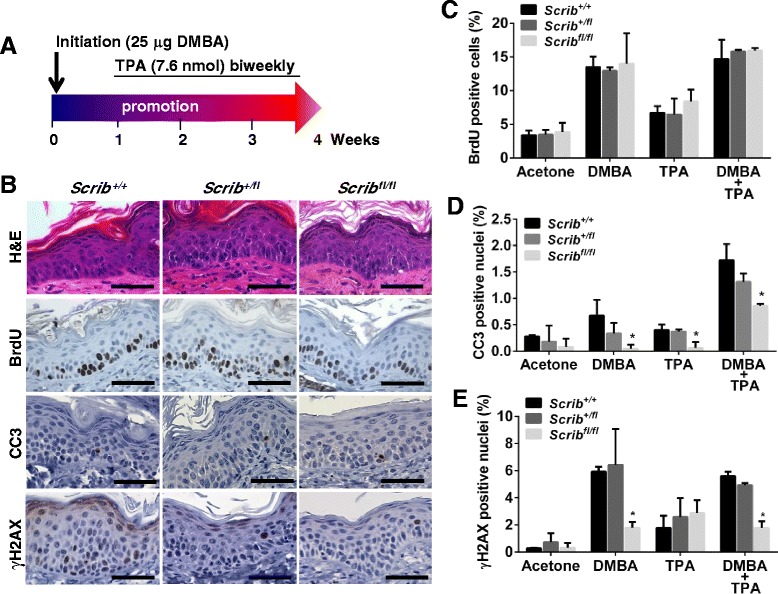
Scrib depletion sensitises the DMBA/TPA-induced pre-neoplastic epidermis to papillomagenesis by inhibiting apoptosis. **a** Diagram illustrating short-term DMBA/TPA administration initiated at 8 weeks of age. **b** Representative H&E images and IHC images for BrdU, CC3, and γH2AX following short-term application of DMBA/TPA in *Scrib* ^*+/+*^, *Scrib* ^*+/fl*^ and *Scrib* ^*fl/fl*^ mice (scale bar = 50 μm, *n* = 3). Quantitation of (**c**) BrdU (**d**) CC3 and (**e**) γH2AX IHC in *Scrib* ^*+/+*^, *Scrib* ^*+/fl*^ and *Scrib* ^*fl/fl*^ epidermis (per 40x magnification field) that have undergone short-term treatment with either acetone, DMBA, TPA or DMBA/TPA (*p ≤ 0.0402, two-way ANOVA with Tukey correction, error bars = SD, *n* = 3). Acetone topical applications served as a control

As *Scrib*^*fl/fl*^ mice were more predisposed to develop DMBA/TPA-induced lesions (Fig. 5b-e), this suggests that Scrib loss may sensitize the epidermis to cutaneous carcinogenesis. To investigate this, we performed a short-term DMBA/TPA study to analyse *Scrib-*deficient epidermis prior to papillomagenesis. To this end, *Wt*, *Scrib*^*+/fl*^ and *Scrib*^*fl/fl*^ mice received a single topical application of DMBA, followed by 4 weeks of TPA treatment (Fig. 7a; *n* = 4–7; mixed gender). Histological analysis of short-term cohorts revealed comparable hyperplastic epidermis associated with epidermal thickening was present in all 3 genotypes (Fig. 7b). We next analysed proliferation rates by quantitating IHC to detect BrdU-positive cells. Surprisingly, Scrib loss does not affect proliferation in DMBA/TPA-induced pre-neoplastic epidermis (Fig. 7b and c). In corroboration, neither DMBA nor TPA treatment alone was sufficient to induce a proliferative advantage in Scrib deficient skin (Fig. 7c and Additional file 4: Figure S4A). Taken together, our findings suggest that although Scrib can suppress proliferation to inhibit the growth of established DMBA/TPA-driven papillomas, Scrib does not affect DMBA/TPA-induced proliferation during the pre-neoplastic state.

The dermal infiltration of immune cells in response to TPA has previously been shown to augment proliferation and promote skin carcinogenesis (reviewed in [50]). Our analysis of mast cells (toluidine blue staining) and macrophages (IHC to detect F4/80) in *Scrib*^*+/+*^ and *Scrib*-deficient epidermis that has undergone short-term treatment with either acetone, DMBA, TPA or DMBA/TPA revealed no difference in the frequency of these immune cell types (Additional file 4: Figure S4B and C). These data indicate that Scrib deletion is not likely to facilitate tumour initiation/growth by mediating inflammatory responses.

Since we observed no changes in proliferation in pre-neoplastic *Scrib*-deficient epidermis, we next investigated the apoptotic index in these mice. Importantly, acetone controls confirmed that Scrib loss is not sufficient to alter CC3-mediated keratinocyte apoptosis. However, compared to *Scrib*^*+/+*^ and *Scrib*^*+/fl*^ mice, the number of CC3-positive cells detected by IHC was significantly decreased in *Scrib*^*fl/fl*^ epidermis treated with either DMBA, TPA or DMBA/TPA for 4 weeks (Fig. **7**b, d and Additional file 5: Figure S5A). Likewise, DMBA treatment (+/− TPA) significantly reduced the number of γH2AX-positive cells in *Scrib*^*fl/fl*^ epidermis (Fig. **7**b, e and Additional file 5: Figure S5B), however the number of p53 and p21 positive nuclei remained unchanged (Additional file 5: Figure S5C-E, and data not shown). Thus, these data suggest that Scrib loss impairs the DMBA/TPA-induced apoptotic response via a p53-independent mechanism, presenting a direct mechanism whereby loss of *Scrib* causes a pre-neoplastic setting in the epidermis.

## Discussion

An impaired epidermal barrier can lead to skin diseases such as psoriasis and eczema, as well as mortality in pre-mature babies. Accordingly, furthering our understanding of the molecular and biochemical events underpinning EPB assembly is paramount to improving our management of skin disorders and pre-mature births. Our data are the first to demonstrate that the polarity regulator Scrib plays a role in the acquisition of the permeability barrier during mouse embryonic development, but is not essential for EPB formation. Our assessment of developmental timing and epidermal barrier components in *Scrib KO* embryos indicates that the transient delay in epidermal barrier acquisition reflects a delay in the maturation of keratinocytes. In support, transgenic mice deficient for the cornified envelope proteins loricrin or envoplakin are also predisposed to delayed barrier formation [51, 52]. Thus, we conclude that although Scrib plays a role in the assembly of the EPB, it is dispensable for envelope formation and barrier function.

As our data demonstrates that *Scrib* is not essential for EPB formation and keratinocyte maturation, this suggests the presence of a compensatory mechanism, as previously proposed for delayed SC maturation following glucocorticoid, estrogen or thyroid hormone reduction and deletion of proliferator-activated receptor (*PPAR*)-α/δ [53–57]. While the rescue of EPB acquisition in *Scrib KO* embryos is likely to reflect the functional redundancy within the polarity network [1, 58, 59] and/or cell intrinsic/extrinsic signalling events mediated by Scrib protein:protein interactions [1–4], future work is necessary to determine how the epidermal development regulation pathways interact. Of note, Scrib has been shown to bind thyroid-stimulating hormone receptor (TSHR) to mediate receptor recycling [60] and hypothyroid mice that harbor a TSHR functional mutation also display a transient delay in SC and EPB formation [53].

We and others have previously demonstrated that Scrib plays a potent tumor suppressive role in mammary, prostate and lung epithelium in the presence of an additional oncogenic event *in vivo* [7, 27, 29, 30]. In agreement, our data is the first to establish that Scrib is an effective epidermal tumor suppressor during initiation and progression of DMBA/TPA-driven cutaneous papillomas, whereas Scrib depletion alone is not sufficient to instigate epidermal tumorigenesis. Mechanistically, we show Scrib loss caused p53-independent apoptosis evasion and reduced the number of cells expressing the DNA damage/pre-apoptotic sensor γH2AX following DMBA/TPA application, thus providing a pro-neoplastic environment in the epidermis. Furthermore, Scrib loss can contribute to papilloma progression by stimulating keratinocyte proliferation. Intriguingly, Iden and colleagues have also reported the polarity protein Par3 is not required for epidermal homeostasis in adult mice, but exerts cell-type dependent pro- and anti-tumorigenic activity during DMBA/TPA-driven skin lesions by regulating cell growth and survival [61], further emphasising the importance of the polarity network during cutaneous carcinogenesis.

Scrib has previously been shown to influence the ability of cells to undergo apoptosis and anoikis in untransformed and transformed cells [27, 30, 33, 62, 63]. Indeed, *SCRIB* depletion in c-MYC-transformed human mammary epithelial cells has been shown to inhibit the Rac-JNK-c-Jun-Bim apoptotic pathway to facilitate c-MYC-driven mammary tumorigenesis [27], and siRNA knockdown of *SCRIB* in HeLa cells infected with the ESEV PDZ-binding motif of the avian influenza A virus NS1 protein reduces virus-induced apoptosis [62]. Our data supports the notion that Scrib mediates apoptosis and in addition, raises the possibility that Scrib is required for the recognition of DNA damage and/or DNA repair given the reduction in the number of γH2AX-positive cells in *Scrib*^*fl/fl*^ DMBA/TPA treated epidermis. It is tempting to speculate that Scrib deletion reduces DMBA-induced DNA damage recognition, thus contributing to the observed apoptosis evasion in *Scrib*^*fl/fl*^ DMBA/TPA-induced skin lesions. Interestingly the polarity protein Par3 has been shown to interact with two regulatory subunits of DNA-dependent protein kinase (DNA-PK) to mediate the repair of DNA double-strand breaks [64], illustrating that the polarity programme directly intersects with the DNA damage repair pathway. It will be important in future studies to fully elucidate the extent to which apoptosis and cell survival are regulated by the polarity network.

Importantly, our findings provide rationale to assess whether Scrib holds prognostic value for skin cancer patients. Indeed, evidence in the literature indicates that human papilloma viruses (HPV) may facilitate the development of SCCs [65, 66] and HPV is reported to target SCRIB for ubiquitin-mediated degradation [67]. Consequently, Scrib loss presents a potential mechanism whereby HPV infection can contribute to the development of cutaneous skin cancers.

In conclusion, we have demonstrated that Scrib mediates EPB acquisition during development and is a potent tumor suppressor during carcinogen-induced skin tumorigenesis and progression that renders cells permissive to DMBA/TPA-induced apoptosis. Apoptosis evasion is a characteristic feature of cancer cells that facilitates tumor initiation and progression, as well as the development of drug resistance [68]. Understanding how a cell gains the ability to resist apoptosis is instrumental to the design of innovative therapies, and our results now indicate that Scrib can regulate this process in skin cancer.

## Methods

### Experimental animals

*EIIaCre* and *K14Cre* transgenic mice that express *Cre* recombinase under the control of the EIIa and K14 promoter have been published previously [37, 69]. *Scrib KO* and *Scrib* floxed mice were generated in-house [7]. All mice were maintained on an FVB/N out bred background and backcrossed at least 9 times. Mice were genotyped from DNA isolated from tail biopsies, as described previously [7]. All animal studies were carried out according to the Peter MacCallum Cancer Centre Animal Experimental Ethics Committee regulations.

### Toluidine blue skin permeability assay

Embryos were harvested at E16.5, E17.5 or E18.5 (*n* = 4–10 per genotype), dehydrated and then rehydrated in a graded series of MeOH in PBS on ice within 5 mins. Embryos were stained with toluidine blue dye (0.1% w/v in PBS) for 10 min and washed in PBS before being imaged under a dissection microscope (Olympus) with an Olympus DP11 camera. Images (1.6x magnifications) were stitched together using Adobe Photoshop CS5 software.

### Embryonic limb development and skeletal analysis

Staining of E17.5 mouse embryos with alcian blue and alizarin red was performed as previously described [70]. Skeletons were photographed in 100 % glycerol, *n* = 3–6 per genotype. Lysotracker analysis of E13.5 mouse limb buds was performed as previously described [71], *n* = 3 per genotype.

### Tissue isolation and histology

Tissue was harvested and fixed for no longer than 24 h in 10 % neutral buffered formaldehyde at 4 °C before being embedded in paraffin and sectioned at 4 μm. Sections were stained with haematoxylin and eosin for histological analysis by a certified pathologist.

### Immunohistochemistry and immunofluorescence staining

Staining was carried out as described previously [7, 29, 72]. Primary antibodies include Cleaved Caspase-3 1:100 (9664, Cell Signalling Technology), PCNA 1:200 (610665, BD Biosciences Pharmingen), Scrib 1:150 (SC1409, Santa Cruz Biotechnology Inc.), Cytokeratin 10 1:100 (ab9026, Abcam), Loricrin 1:1000 (PRB-145P, Covance), BrdU 1:150 (#347-580, BD Biosciences) ZO-1 1:50 (61–7300, Thermo Scientific), γH2AX 1:300 (#NB100-2280, Novus Biologicals), p-ERK1/2 (Thr 202/Tyr204) 1:150 (4376, Cell Signaling Technology), F4/80 1:200 (MCA497G, AbD Serotec), p53 1:200 (SC-6243, Santa Cruz), p21 1:100 (SC-397, Santa Cruz), CK5 1:1000 (PRB-160P, Covance) and CK6 1:500 (PRB-169P, Covance). Negative control slides were run without primary antibody, and positive control slides were incorporated. IHC/IF scoring was performed either manually (untreated and short-term DMBA/TPA treatment analysis) or using Molecular Devices MetaMorph 6.3 software (long-term DMBA/TPA treatment analysis) from 6–10 images per mouse (×40 magnification, BX-61 Olympus microscope). All experiments were performed using embryonic flank epidermis, adult dorsal skin or DMBA/TPA-driven skin lesions as indicated.Mast cell stainingFFPE sections were rehydrated and incubated in 0.5% aqueous potassium permanganate for 2 minutes, rinsed in distilled water and incubated in 2% aqueous potassium metabisulphite for 1 minute. After washing in distilled water, sections were incubated for 5 min in acidified toluidine blue solution (0.25 ml glacial acetic acid, 0.02g toluidine blue in 99.75 ml distilled water), rinsed in distilled water and dehydrated prior being coverslipped.

### Ultrastructural analysis

Tissue was fixed in 2 % paraformaldehyde, 2.5 % glutaraldehyde in 0.08 M Sorensen’s phosphate buffer (PBS) for 2 h and washed in PBS before being immersed in 2 % osmium tetroxide in PBS and dehydrated through a graded series of alcohols, two acetone rinses and embedding in Spurrs resin. Sections approximately 80 nm thick were cut with a diamond knife (Diatome, Switzerland) on an Ultracut-S ultramicrotome (Leica, Mannheim, Germany) and contrasted with uranyl acetate and lead citrate. Images were captured with a Megaview II cooled CCD camera (Soft Imaging Solutions, Olympus) using a JEOL 1011 transmission electron microscope (TEM).

### Quantitation of epidermal layer thickness

Using Molecular Devices MetaMorph 6.3 software, epidermal thickness (i.e. the distance between the basement membrane and epidermal surface) was measured 3 independent times on 10 H&E images (4 μm thick) for each embryo. SC and SG thickness was measured 3 independent times on 5 toluidine blue stained ultramicrotome sections (80 nm thick) for each embryo. Scored images of flank epidermis were taken at 40x magnification (*n* = 3 per genotype/time point).

### QRT-PCR analysis

RNA was isolated using TRizol (Invitrogen), TURBO DNase treated (Ambion), and reverse transcribed with Superscript III (Invitrogen). Amplification of cDNA was performed by the StepOne-Plus Real-Time PCR System (Applied Biosystems). Samples were normalized to *Gapdh*, and Scrib mRNA fold change was calculated as described previously [7]. Lgr5 mRNA expression was detected as described previously [73].

### DMBA/TPA administration

Chemical skin carcinogenesis was initiated at 8 weeks of age on shaved dorsal skin by a single topical treatment with 25 μg DMBA (Sigma Aldrich) in 50 μl acetone. One week post-DMBA initiation, biweekly topical application of 7.6 nmol TPA (Sigma Aldrich) in 150 μl acetone were performed for; (i) 20 week, with an additional 6 weeks without TPA (long-term study, *n* = 12–16 per genotype) or (ii) 4 weeks (short-term, *n* = 4–7 per genotype). Mice were shaved, imaged and lesion volume recorded on a weekly basis and animals harbouring lesions >1 cm in diameter were sacrificed in accordance with our ethical limit.

### Statistical analysis

Statistical analysis was performed using GraphPad Prism 6 software using either a two-tailed unpaired *t*-test (95 % confidence interval) or a two-way ANOVA (with Tukey correction) as indicated. H&E, IHC staining and qRT-PCR quantitation was analysed statistically for at least 3 mice per genotype. QRT-PCR reactions were performed in triplicate, and a *P* value less than 0.05 was considered statistically significant. Error bars represent standard deviation (SD) or standard error of the mean (SEM) as indicated.

## Additional files

Additional file 1: Figure S1. Cell polarity is not deregulated in *Scrib* KO embryonic epidermis. (A) IF to detect pan-Dlg (*green*) and DAPI (*blue*) and (B) IF to detect E-cadherin (*red*) and DAPI (*blue*) in *Scrib* Wt, Het and KO embryonic epidermis at E17.5 (*n* = 3, scale bar = 50 μm, dashed line represents basement membrane). (PPTX 539 kb) Additional file 2: Figure S2. Scrib is not essential for epidermal homeostasis in adult mice. (A) qRT-PCR to detect *Lgr5* confirmed *Scrib* ^+/+^, *Scrib* ^*+/*^^*fl*^and *Scrib* ^*fl/fl*^ adult dorsal epidermis display a similar level of *Lgr5* mRNA transcript expression (*P* ≥ 0.1013, unpaired *t*-test, error bars = SD, *n* = 3). (B) IHC to detect cytokeratin 6 (K6) in *Scrib* ^+/+^, *Scrib* ^*+/*^^*fl*^ and *Scrib* ^*fl/fl*^ adult dorsal epidermis (scale bar = 50 μm, *n* = 3). (C) IF to detect cytokeratin 5 (K5, *green*), cytokeratin 10 (K10, *red*) and DAPI (*blue*) in Scrib^+/+^, *Scrib* ^*+/*^^*fl*^ and *Scrib* ^*fl/fl*^ adult dorsal epidermis (scale bar = 50 μm, *n* = 3). Adult dorsal epidermis was harvested from mice 100 days old. (PPTX 755 kb) Additional file 3: Figure S3. Analysis of *Scrib*-deficient DMBA/TPA-driven epidermal lesions. (A) Kaplan Meier survival plot for DMBA/TPA treated *Scrib* ^*+/+*^ *, Scrib* ^*+/fl*^ and *Scrib* ^*fl/fl*^ mice. The average survival of *Scrib* ^*fl/fl*^ mice (23.5 weeks post-DMBA) was comparable to *Scrib* ^*+/+*^ and *Scrib* ^*+/fl*^ mice (24.5 and 24.9 weeks post-DMBA respectively). No statistical difference in survival was observed between genotypes (χ^2^ = 0.029 – 2.15, df = 1, *P* ≥ 0.1426, Log-rank Mantel-Cox test, *n* = 12–16). (B) qRT-PCR for *Scrib* mRNA confirmed a significant reduction in *Scrib* ^*+/fl*^ and *Scrib* ^*fl/fl*^ volume-matched (40 – 80 mm^3^) early benign papillomas compared to *Scrib* ^*+/+*^ lesions (**p* < 0.0001, unpaired *t*-test, *n* = 3). (C) IF to detect Scrib (*green*) and DAPI (*blue*) in *Scrib* ^*+/+*^, *Scrib* ^*+/fl*^ and *Scrib* ^*fl/fl*^ DMBA/TPA-induced size-matched benign papillomas (*n* = 3, scale bar = 50 μm, insert 1–3 scale bar = 10 μm). Representative p-ERK IHC images (D) and quantitation (E) from *Scrib* ^*+/+*^, *Scrib* ^*+/fl*^ and *Scrib* ^*fl/fl*^ DMBA/TPA-induced size-matched benign papillomas (scale bar = 50 μm, *P* ≥ 0.5919, unpaired *t*-test, error bars = SD, *n* = 3). (F) IF to detect ZO-1 (*green*), E-cadherin (*red*) and DAPI (*blue*) in *Scrib* ^*+/+*^, *Scrib* ^*+/fl*^ and *Scrib* ^*fl/fl*^ DMBA/TPA-induced size-matched benign papillomas (*n* = 3, scale bar = 50 μm). (PPTX 1616 kb) Additional file 4: Figure S4. Analysis of proliferation and immune response to short-term DMBA and/or TPA treatment in *Scrib*-deficient mice. (A) IHC to detect BrdU in *Scrib* ^*+/+*^, *Scrib* ^*+/fl*^ and *Scrib* ^*fl/fl*^ dorsal epidermis that has undergone short-term treatment with either acetone, DMBA or TPA. (B) IHC to detect F4/80 and identify macrophages and (C) toluidine blue staining to detect mast cells in *Scrib* ^*+/+*^, *Scrib* ^*+/fl*^ and *Scrib* ^*fl/fl*^ dorsal epidermis that has undergone short-term treatment with either acetone, DMBA, TPA or DMBA/TPA. Scale bar = 50 μm, *n* = 3. (PPTX 4121 kb) Additional file 5: Figure S5. Analysis of short-term DMBA and/or TPA induced apoptosis in *Scrib*-deficient mice. IHC to detect (A) CC3 and (B) γH2AX in *Scrib* ^*+/+*^, *Scrib* ^*+/fl*^ and *Scrib* ^*fl/fl*^ dorsal epidermis that has undergone short-term treatment with either acetone, DMBA or TPA (scale bar = 50 μm, *n* = 3). (C) Representative IHC images to detect p53 and p21 (scale bar = 50 μm, *n* = 3) and quantitation of p53 (D) and p21 (E) IHC (*P* ≥ 0.4776, unpaired *t*-test, error bars = SD, *n* = 3) in *Scrib* ^*+/+*^, *Scrib* ^*+/fl*^ and *Scrib* ^*fl/fl*^ dorsal epidermis that has undergone short-term treatment with DMBA/TPA. (PPTX 2730 kb)

## Abbreviations

AJ: Adheren’s Junction
DMBA: 7,12-Dimethylbenz(a)anthracene
EPB: Epidermal permeability barrier
CC3: Cleaved Caspase-3
K: Cytokeratin
H&E: Haematoxylin and eosin
IF: Immunofluorescence
IHC: Immunohistochemistry
QRT-PCR: Quantitative real time polymerase chain reaction
SC: Stratum corneum
SCC: Squamous cell carcinoma
SD: Standard deviation
SEM: Standard error of the mean
SG: Stratum granulosum
TPA: 12-*O*-Tetradecanoylphorbol-13-acetate
