# Supplementary figures and images for: The polarity protein Scrib mediates epidermal development and exerts a tumor suppressive function during skin carcinogenesis
Source: Mol Cancer. 2015 Sep 17;14:169. doi: 10.1186/s12943-015-0440-z (PMC4574215; doi:10.1186/s12943-015-0440-z)

## Slide 1
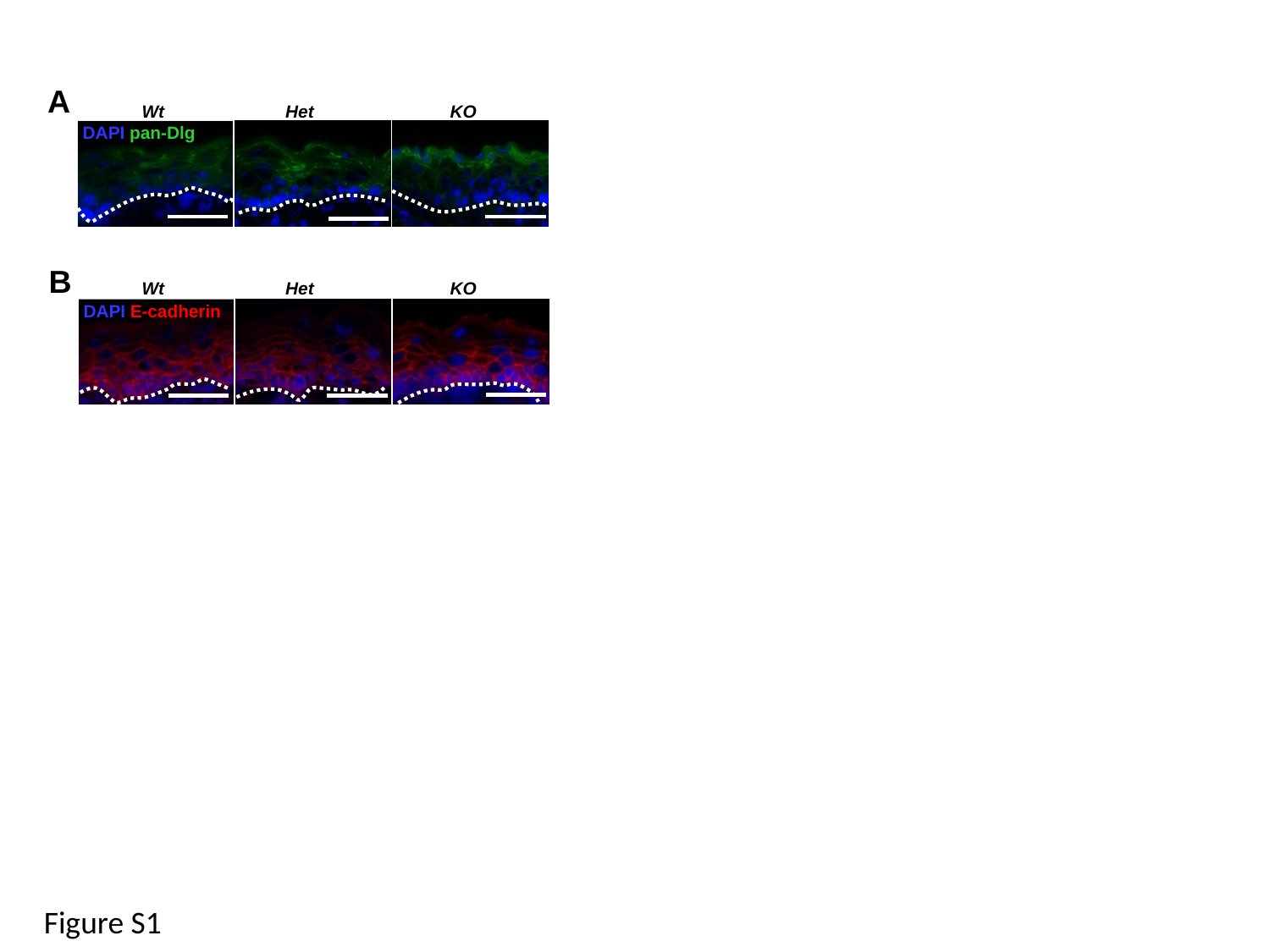

A
 Wt Het KO
DAPI pan-Dlg
B
 Wt Het KO
DAPI E-cadherin
Figure S1

Supplement: Additional file 1: Figure S1. — Cell polarity is not deregulated in Scrib KO embryonic epidermis. (A) IF to detect pan-Dlg (green) and DAPI (blue) and (B) IF to detect E-cadherin (red) and DAPI (blue) in Scrib Wt, Het and KO embryonic epidermis at E17.5 (n = 3, scale bar = 50 μm, dashed line represents basement membrane). (PPTX 539 kb) [file 12943_2015_440_MOESM1_ESM.pptx]

## Slide 1
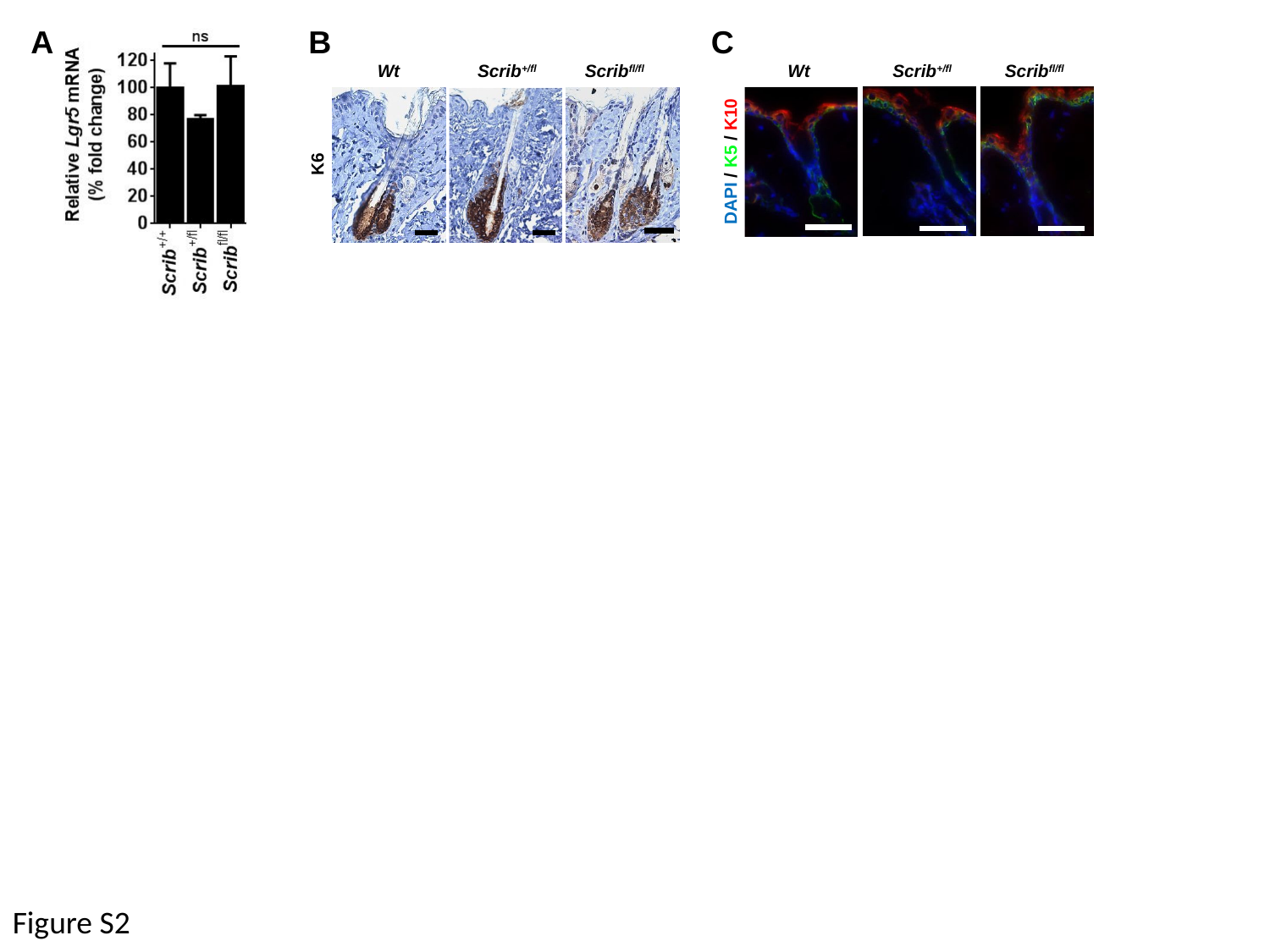

A
B
C
 Wt Scrib+/fl Scribfl/fl
Wt Scrib+/fl Scribfl/fl
K6
DAPI / K5 / K10
Figure S2

Supplement: Additional file 2: Figure S2. — Scrib is not essential for epidermal homeostasis in adult mice. (A) qRT-PCR to detect Lgr5 confirmed Scrib +/+, Scrib +/ fland Scrib fl/fl adult dorsal epidermis display a similar level of Lgr5 mRNA transcript expression (P ≥ 0.1013, unpaired t-test, error bars = SD, n = 3). (B) IHC to detect cytokeratin 6 (K6) in Scrib +/+, Scrib +/ fl and Scrib fl/fl adult dorsal epidermis (scale bar = 50 μm, n = 3). (C) IF to detect cytokeratin 5 (K5, green), cytokeratin 10 (K10, red) and DAPI (blue) in Scrib+/+, Scrib +/ fl and Scrib fl/fl adult dorsal epidermis (scale bar = 50 μm, n = 3). Adult dorsal epidermis was harvested from mice 100 days old. (PPTX 755 kb) [file 12943_2015_440_MOESM2_ESM.pptx]
