# Supplementary material for: The polarity protein Scrib mediates epidermal development and exerts a tumor suppressive function during skin carcinogenesis
Source: Mol Cancer. 2015 Sep 17;14:169. doi: 10.1186/s12943-015-0440-z (PMC4574215; doi:10.1186/s12943-015-0440-z)
Supplement: Additional file 3: Figure S3. — Analysis of Scrib-deficient DMBA/TPA-driven epidermal lesions. (A) Kaplan Meier survival plot for DMBA/TPA treated Scrib +/+ , Scrib +/fl and Scrib fl/fl mice. The average survival of Scrib fl/fl mice (23.5 weeks post-DMBA) was comparable to Scrib +/+ and Scrib +/fl mice (24.5 and 24.9 weeks post-DMBA respectively). No statistical difference in survival was observed between genotypes (χ2 = 0.029 – 2.15, df = 1, P ≥ 0.1426, Log-rank Mantel-Cox test, n = 12–16). (B) qRT-PCR for Scrib mRNA confirmed a significant reduction in Scrib +/fl and Scrib fl/fl volume-matched (40 – 80 mm3) early benign papillomas compared to Scrib +/+ lesions (*p < 0.0001, unpaired t-test, n = 3). (C) IF to detect Scrib (green) and DAPI (blue) in Scrib +/+, Scrib +/fl and Scrib fl/fl DMBA/TPA-induced size-matched benign papillomas (n = 3, scale bar = 50 μm, insert 1–3 scale bar = 10 μm). Representative p-ERK IHC images (D) and quantitation (E) from Scrib +/+, Scrib +/fl and Scrib fl/fl DMBA/TPA-induced size-matched benign papillomas (scale bar = 50 μm, P ≥ 0.5919, unpaired t-test, error bars = SD, n = 3). (F) IF to detect ZO-1 (green), E-cadherin (red) and DAPI (blue) in Scrib +/+, Scrib +/fl and Scrib fl/fl DMBA/TPA-induced size-matched benign papillomas (n = 3, scale bar = 50 μm). (PPTX 1616 kb) [file 12943_2015_440_MOESM3_ESM.pptx]

## Slide 1
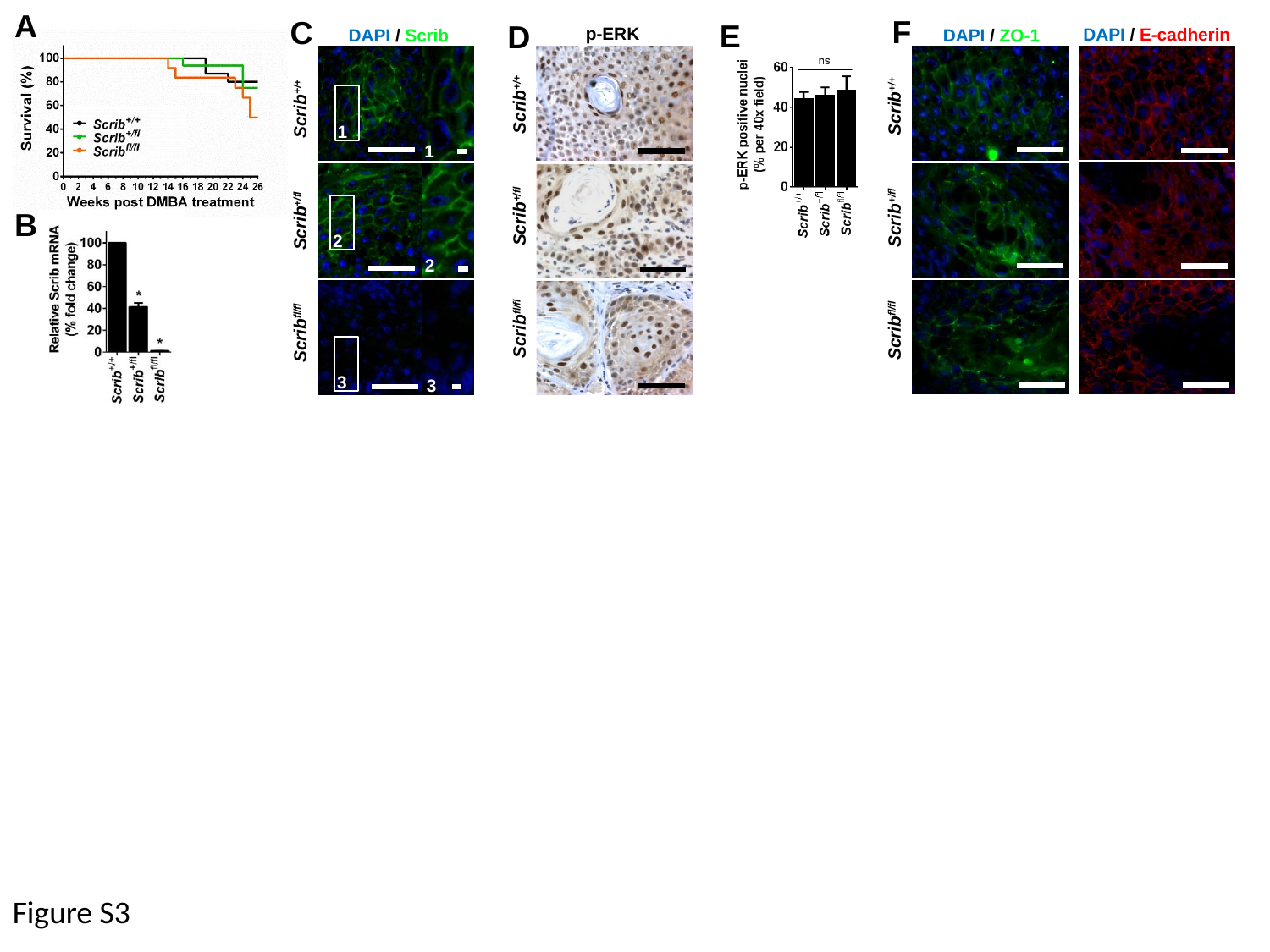

A
F
C
E
D
 p-ERK
DAPI / E-cadherin
DAPI / Scrib
DAPI / ZO-1
Scribfl/fl Scrib+/fl Scrib+/+
Scribfl/fl Scrib+/fl Scrib+/+
Scribfl/fl Scrib+/fl Scrib+/+
1
1
B
2
2
3
3
Figure S3
