# Supplementary material for: The polarity protein Scrib mediates epidermal development and exerts a tumor suppressive function during skin carcinogenesis
Source: Mol Cancer. 2015 Sep 17;14:169. doi: 10.1186/s12943-015-0440-z (PMC4574215; doi:10.1186/s12943-015-0440-z)
Supplement: Additional file 4: Figure S4. — Analysis of proliferation and immune response to short-term DMBA and/or TPA treatment in Scrib-deficient mice. (A) IHC to detect BrdU in Scrib +/+, Scrib +/fl and Scrib fl/fl dorsal epidermis that has undergone short-term treatment with either acetone, DMBA or TPA. (B) IHC to detect F4/80 and identify macrophages and (C) toluidine blue staining to detect mast cells in Scrib +/+, Scrib +/fl and Scrib fl/fl dorsal epidermis that has undergone short-term treatment with either acetone, DMBA, TPA or DMBA/TPA. Scale bar = 50 μm, n = 3. (PPTX 4121 kb) [file 12943_2015_440_MOESM4_ESM.pptx]

## Slide 1
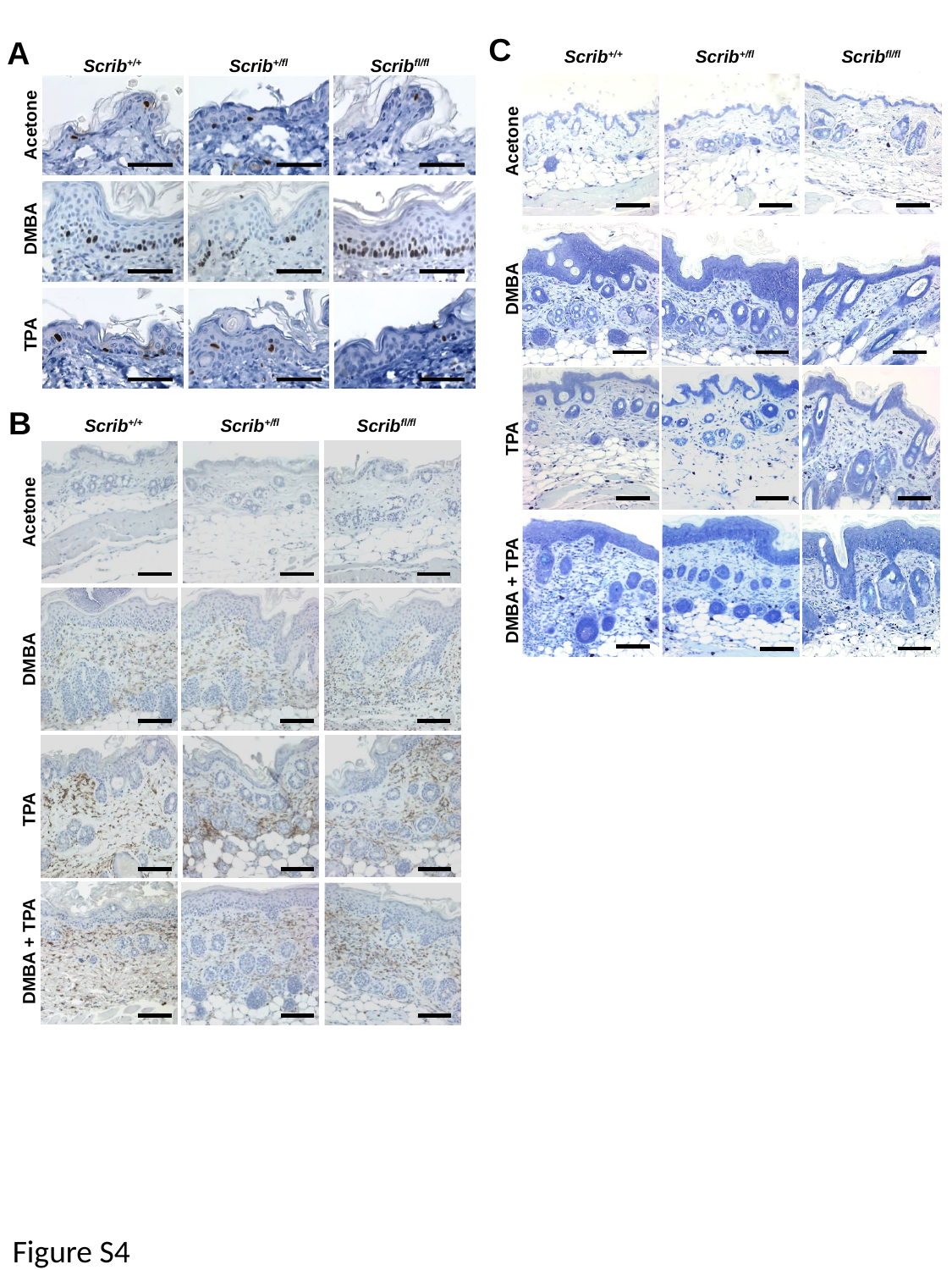

C
A
Scrib+/+ Scrib+/fl Scribfl/fl
Scrib+/+ Scrib+/fl Scribfl/fl
TPA DMBA Acetone
DMBA + TPA TPA DMBA Acetone
B
Scrib+/+ Scrib+/fl Scribfl/fl
DMBA + TPA TPA DMBA Acetone
Figure S4
