# Supplementary material for: The polarity protein Scrib mediates epidermal development and exerts a tumor suppressive function during skin carcinogenesis
Source: Mol Cancer. 2015 Sep 17;14:169. doi: 10.1186/s12943-015-0440-z (PMC4574215; doi:10.1186/s12943-015-0440-z)
Supplement: Additional file 5: Figure S5. — Analysis of short-term DMBA and/or TPA induced apoptosis in Scrib-deficient mice. IHC to detect (A) CC3 and (B) γH2AX in Scrib +/+, Scrib +/fl and Scrib fl/fl dorsal epidermis that has undergone short-term treatment with either acetone, DMBA or TPA (scale bar = 50 μm, n = 3). (C) Representative IHC images to detect p53 and p21 (scale bar = 50 μm, n = 3) and quantitation of p53 (D) and p21 (E) IHC (P ≥ 0.4776, unpaired t-test, error bars = SD, n = 3) in Scrib +/+, Scrib +/fl and Scrib fl/fl dorsal epidermis that has undergone short-term treatment with DMBA/TPA. (PPTX 2730 kb) [file 12943_2015_440_MOESM5_ESM.pptx]

## Slide 1
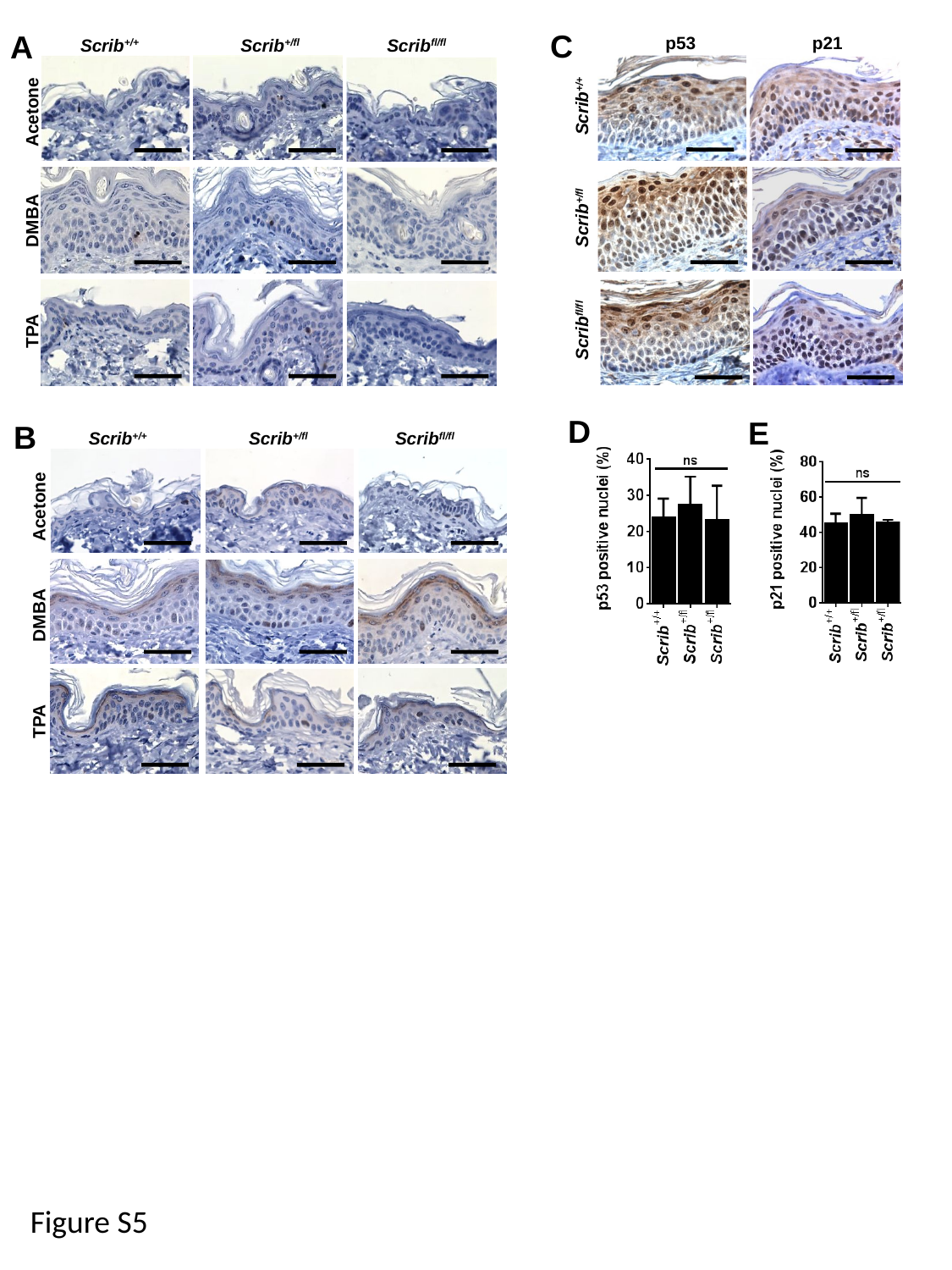

C
A
p53 p21
Scrib+/+ Scrib+/fl Scribfl/fl
Scribfl/fl Scrib+/fl Scrib+/+
TPA DMBA Acetone
D
E
B
Scrib+/+ Scrib+/fl Scribfl/fl
TPA DMBA Acetone
Figure S5
